# *In Vitro* Culture of Functionally Active Buffalo Hepatocytes Isolated by Using a Simplified Manual Perfusion Method

**DOI:** 10.1371/journal.pone.0118841

**Published:** 2015-03-19

**Authors:** Santanu Panda, Sonu Bisht, Dhruba Malakar, Ashok K. Mohanty, Jai K. Kaushik

**Affiliations:** Animal Biotechnology Centre, National Dairy Research Institute, Karnal, 132001, Haryana, India; Montana State University, UNITED STATES

## Abstract

**Background:**

In farm animals, there is no suitable cell line available to understand liver-specific functions. This has limited our understanding of liver function and metabolism in farm animals. Culturing and maintenance of functionally active hepatocytes is difficult, since they survive no more than few days. Establishing primary culture of hepatocytes can help in studying cellular metabolism, drug toxicity, hepatocyte specific gene function and regulation. Here we provide a simple *in vitro* method for isolation and short-term culture of functionally active buffalo hepatocytes.

**Results:**

Buffalo hepatocytes were isolated from caudate lobes by using manual enzymatic perfusion and mechanical disruption of liver tissue. Hepatocyte yield was (5.3±0.66)×10^7^ cells per gram of liver tissue with a viability of 82.3±3.5%. Freshly isolated hepatocytes were spherical with well contrasted border. After 24 hours of seeding onto fibroblast feeder layer and different extracellular matrices like dry collagen, matrigel and sandwich collagen coated plates, hepatocytes formed confluent monolayer with frequent clusters. Cultured hepatocytes exhibited typical cuboidal and polygonal shape with restored cellular polarity. Cells expressed hepatocyte-specific marker genes or proteins like albumin, hepatocyte nuclear factor 4α, glucose-6-phosphatase, tyrosine aminotransferase, cytochromes, cytokeratin and α1-antitrypsin. Hepatocytes could be immunostained with anti-cytokeratins, anti-albumin and anti α1-antitrypsin antibodies. Abundant lipid droplets were detected in the cytosol of hepatocytes using oil red stain. *In vitro* cultured hepatocytes could be grown for five days and maintained for up to nine days on buffalo skin fibroblast feeder layer. Cultured hepatocytes were viable for functional studies.

**Conclusion:**

We developed a convenient and cost effective technique for hepatocytes isolation for short-term culture that exhibited morphological and functional characteristics of active hepatocytes for studying gene expression, regulation, hepatic genomics and proteomics in farm animals.

## Introduction

Liver is the main organ for metabolism, biotransformation of drugs and xenobiotics and storage of different biomolecules. Understanding of liver metabolism in ruminants is crucial to improve animal health, productivity and reproduction, which in turn has great impact on animal production [1]. Primary hepatocytes are well representative of *in vivo* hepatocytes and can be used for studying metabolic activities [2] as well as to explore pharmacological properties of drugs and xenobiotics [3, 4]. Primary hepatocytes are also useful precursors for artificial liver development [5, 6] and cell transplantation studies for treatment of acute and chronic liver failure [7–9]. Most of the work on hepatocytes has been done on human or animal systems to study liver function, liver pathophysiology and disease conditions. Farm animals play an important role in the food system. Production, reproduction and animal health are directly linked to feed utilization efficiency and biotransformation taking place in liver. However, no ruminant specific immortal hepatic cell line is available to study the liver function in farm animals. Under such scenario, primary cell culture is the most viable material for studying metabolism, pharmacology of drugs and xenobiotics metabolism, gene expression and regulation to understand the liver function. Hepatocytes isolation and culture is a crucial step to study liver metabolism as well as biotransformation reactions of nitrogen and sulphur containing veterinary drugs and pesticides, their interactions with liver enzymes and other drug binding proteins. Understanding of pharmacological and toxicological properties of various drugs and xenobiotics or even feed components may be exploited to manage animal health for improving milk and meat production in farm animals.

Liver parenchyma is surrounded by fibrillar network of collagen that is strengthened by extracellular matrix (ECM) like elastin, heparin sulphate, proteoglycan, laminin and fibronectin. Hepatocytes are specialized epithelium with distinct apical (bile canalicular) and basal (sinusoidal) surface representing 65% of total cell number and 78% of liver volume. This discrepancy between cell number and volume is due to larger size of hepatocytes than other non-parenchymal cells. Only 6% of liver volume consists of non-parenchymal cells like endothelial cells and Kuffer cells lining the sinusoids, fat storing stellate or Ito cells and natural killer lymphocytes. The remaining 16% space is occupied by intercellular space, i.e sinusoidal lumen, biliary canaliculi and Disse space (for review, see [10]).

Successful isolation and culture of hepatocytes has been a challenging job for many decades. Techniques of hepatocytes isolation started in rat liver [11] and subsequently applied to human and several other species. The technique was refined by Berry and Friend [12] and further by Seglen [13] by using two steps perfusion method employing Ca^2+^ chelator ethylene glycol tetra acetate (EGTA) and collagenase. Seglen’s two steps perfusion method is the gold standard for hepatocytes isolation and several reports were published on isolation of hepatocytes in ruminants using modified or similar method [3, 14–17]. These methods require extended processing time and costly chemicals or equipments. Hepatocytes suffer with limited life span and quick loss of liver specific function in culture condition. Therefore, it is crucial to use an easy method for quick recovery of functionally active hepatocytes with high purity. Here we report an easy and cost effective method for hepatocyte isolation with adequate cell yield, good viability and high purity. The cultured cells showed morphological and functional characteristics of hepatocytes in terms of expression of several hepatocyte specific marker genes or proteins. The method relies on manual perfusion by using a 50 ml aseptic syringe to obtain sufficient quantity of hepatocytes. Furthermore, by using skin fibroblasts as feeder layer the hepatocytes could be grown for five days with life span of nine-ten days in culture.

## Results

### Hepatocytes yield, purity and viability

The modified procedure of Seglen’s method [13] which used combination of enzymatic and mechanical disruption steps has been developed to isolate hepatocytes from caudate lobe of buffalo liver for retrieving functionally viable and pure cells. We got a cell yield of (5.3 ± 0.7)×10^7^ cells per gram of liver tissue with (82.3 ± 3.5) % (*n* = 3) cell viability as assessed by standard trypan blue dye exclusion method. The cellular purity was about 99% after three washing steps, which were performed by centrifugation at 50×g for 1 minute each time to remove RBC and other contaminating cells. Centrifugation yielded hapatocytes with high purity and can be used to replace the standard Percoll based separation of cells. Our results are comparable to that reported for rat hepatocytes by Seglen’s method [13] with cellular yield of (5.0 ± 2.7)×10^7^ cells/g liver tissue with 88% viability. Several studies reported improved recovery of hepatocytes up to 10^7–8^ cells in cattle [1, 17], 2×10^10^ cells at 97% viability and 99% purity in pig [18, 19], 10^9^ cells with 60% viability in canine [19] and 2×10^7–9^ cells in human [20, 21]. Our results on buffalo hepatocytes were similar to that obtained on rat, cattle and human, however, cell yield and viability in case of pig was significantly higher. In case of canines, the cell yield was high but viability was low. In case of non-perfusion method for cattle hepatocytes, the collagenase was used at a concentration of 500 ng/ml (0.0005% w/v) [17], which was 2–3 orders lower than used in perfusion based methods requiring collagenase at 0.05–0.5% (w/v). The obtained cell purity was lower and required further purification of hepatocytes by Percoll [17]. We used perfusion with a collagenase concentration of 500 ng/ml in combination with mechanical disruption to achieve high purity, descent yield and viability of buffalo hepatocytes. The results are comparable to that achieved by perfusion methods utilizing much higher collagenase concentration.

### Morphology and proliferation of isolated hepatocytes

**Fig 1:**
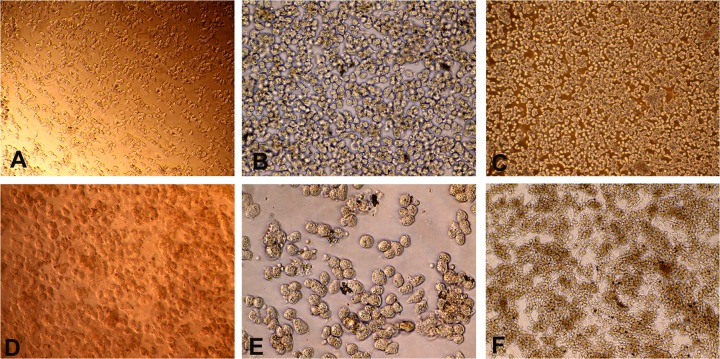
Primary buffalo hepatocyte culture on different extracellular matrices. Panel A: Fresh hepatocytes on DCC (100X); B: Fresh hepatocytes on DCC (200X); C: Hepatocyes after 24 h on DCC (100X); D: Hepatocytes after 3 days on Matrigel (200X); E: Hepatocytes after 7 days in sandwich collagen matrix (400X); F: 5 days old hepatocytes grown on feeder layer of buffalo skin fibroblast (200X).

Inverted phase contrast microscopy showed freshly isolated undamaged hepatocytes to be bright, translucent and spherical in shape with characteristic well contrasted border (Fig. 1), whereas the damaged hepatocytes formed bleb in the plasma membrane. Cells exhibited polygonal shape with centrally located one or two nucleus after 24 hours of incubation on various ECMs like dry collagen coat, matrigel and sandwich collagen gel (Fig. 1). The isolated hepatocytes again aggregated into clusters and established cell-cell interaction and cellular polarity. Hepatocytes seeded at a concentration of 3.5×10^6^ viable cells/ml in 60 mm diameter culture dishes could be grown for 5–7 days on various ECMs. On feeder layer of skin fibroblast, most of the hepatocytes attached within 2 hours and formed isolated clumps (Fig. 1F).

**Fig 2:**
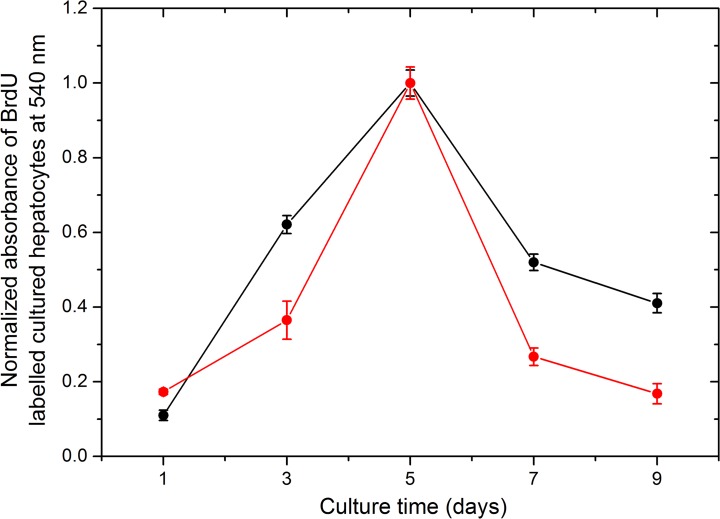
Hepatocyte growth on fibroblast feeder layer. Hepatocyte proliferation curve represented by the absorbance at 540 nm of BrdU-labelled hepatocytes at different days of culture.

Our results on cell proleferation determined by 5-bromo-2'-deoxyuridine (BrdU) incorporation assay suggested that hepatocytes divided up to 5^th^ day after seeding and then growth receded in both cases, with or without feeder layer (Fig. 2). Significant change in the growth rate of hepatocytes was observed in between the cultures with or without feeder layer. The normalized curves suggested higher rate of hepatocyte growth on 3^rd^ day and after reaching maximal growth (5–9 days) on feeder layer in comparison to cells growing without feeder layer. The decrease in hepatocyte growth rate in culture without feeder layer was steeper after 5^th^ day in comparison to cells growing on feeder layer. The data indicated that BrdU incorporation on days 7–9 was similar to that at day 1 when feeder layer was not used. On the other hand, feeder layer helped in the growth of hepatocytes at 7–9 days as indicated by higher level of BrdU incorporation on these days.

### Functional characterization of buffalo hepatocytes

Functional assessment of the cultured hepatocytes was performed by expression analysis of marker genes by using reverse transcriptase polymerase chain reaction (RT-PCR), Western-blotting, immunostaining and oil red staining for detection of fat droplets.

**Fig 3:**
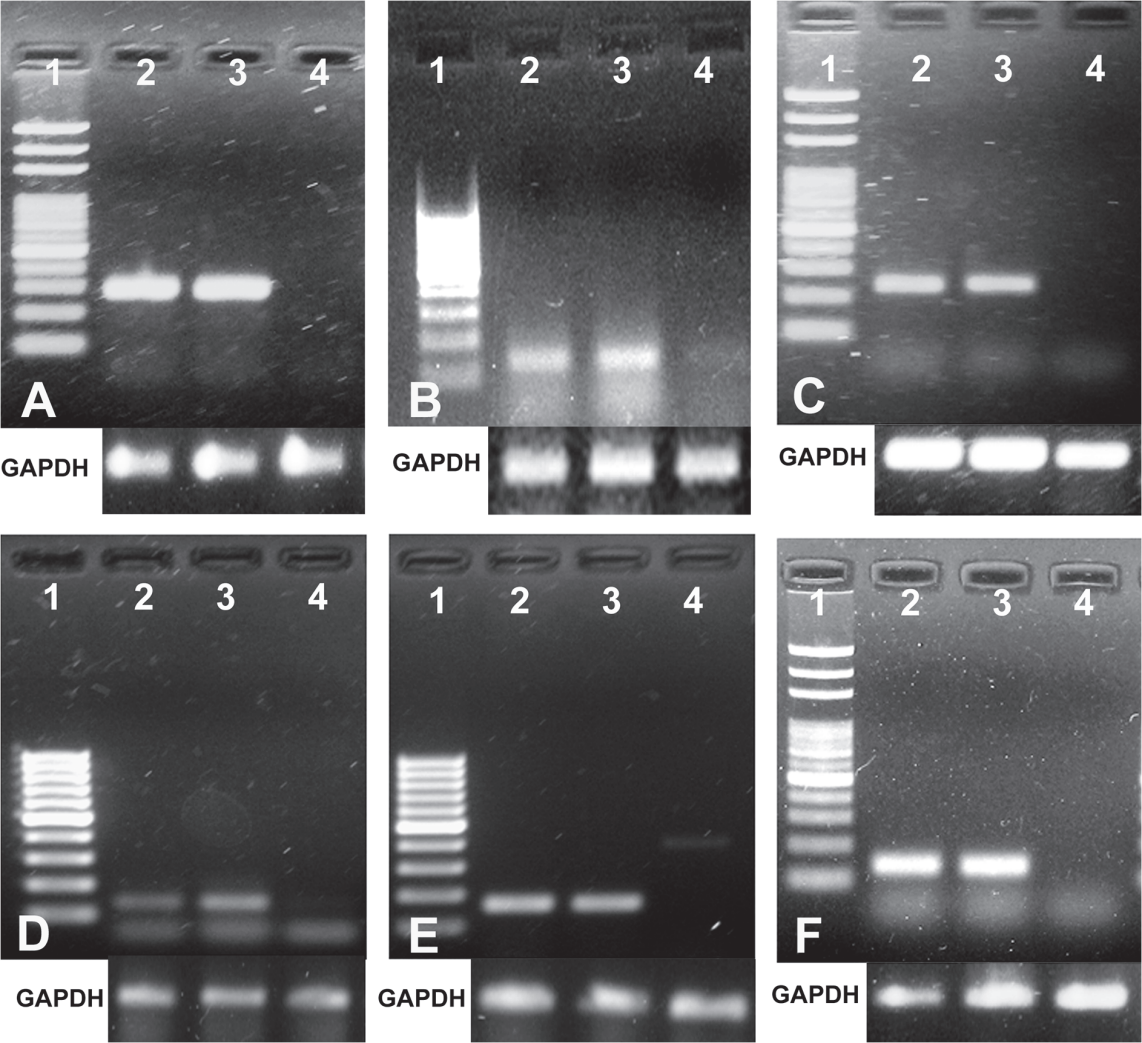
Agarose gel electrophoresis of RT-PCR products of hepatocyte-specific marker genes expressed in 5 days old cultured hepatocytes. Panel A shows 293 bp amplicon of albumin; B—130 bp amplicon of hepatocyte nuclear factor 4α; C—240 bp amplicon of glucose-6-phosphatase; D—136 bp amplicon of CYP1A1; E—164 bp amplicon of CYP3A4; F—142 bp amplicon of tyrosine aminotransferase. Lane 1: 100 bp ladder; Lane 2: RT-PCR of liver tissue (positive control) by using the gene-specific primers; Lane 3: RT-PCR of respective genes from cultured buffalo hepatocytes; Lane 4: RT-PCR from skin fibroblasts (negative control). Amplification of Glyceraldehyde 3–phosphate dehydrogenase (GAPDH) was used as housekeeping gene.

**Expression analysis of hepatocyte-specific marker genes**. Expression of hepatocyte specific marker genes was analysed on 5^th^ day of cultured cells by RT-PCR. Amplification of correct size amplicons by using gene-specific primers designed for albumin, tyrosine aminotransferase, glucose-6-phosphatase, hepatocyte nuclear factor 4α (HNF-4α), cytochrome P450 (CYP1A1 and CYP3A4) suggested the expression of these genes in the cultured hepatocytes on 5^th^ day. There was no expression of these marker genes in the buffalo skin fibroblast cells, which were used as negative control. Robust amplification of albumin cDNA suggested normal albumin secreting property of the cultured cells. Tyrosine aminotransferase and glucose-6–phosphatase that are important marker enzymes of liver cells for amino acid and carbohydrate metabolism, respectively, were also detected in abundance at transcript level. Genes encoding for CYP1A1, CYP3A4, and HNF-4α were also expressed in hepatocytes, suggesting the maintenance of important functional aspects of hepatocytes in the cultured cells (Fig. 3).

**Fig 4:**
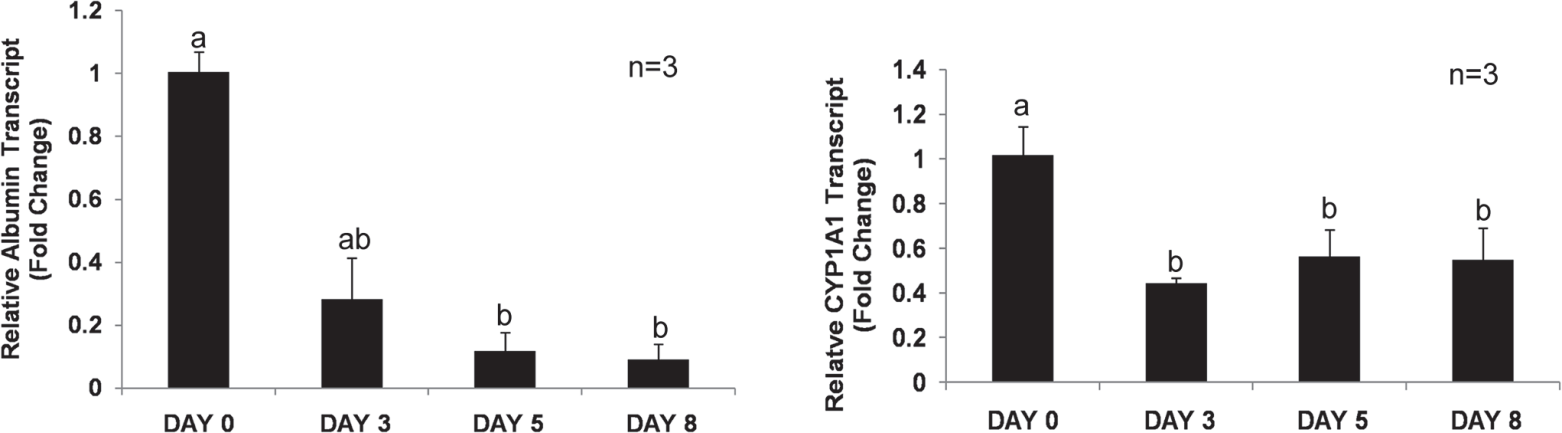
Real-time quantitative PCR analysis of albumin and CYP1A1 gene expression in cultured hepatocytes. Panel A shows relative transcript level of albumin and, Panel B shows CYP1A1 relative transcript level expressed as fold change.

Real time quantitative PCR of albumin gene revealed decreased transcripts level of 0.28, 0.11 and 0.09 folds on 3^rd^, 5^th^ and 8^th^ day of hepatocytes culture, respectively. Significant difference (Students’ paired t-test at p≤0.05) in albumin transcript level was observed between fresh, 3^rd^ and 5^th^ day of culture. CYP1A1 expression was also downregulated to 0.44, 0.56 and 0.54 fold on 3^rd^, 5^th^ and 8^th^ days, respectively, in comparison to fresh hepatocytes (Fig. 4). These results suggested a larger decrease in the expression of albumin gene in comparison to CYP1A1, whose expression was more or less unchanged during 3–8 days of the culture.

**Fig 5:**
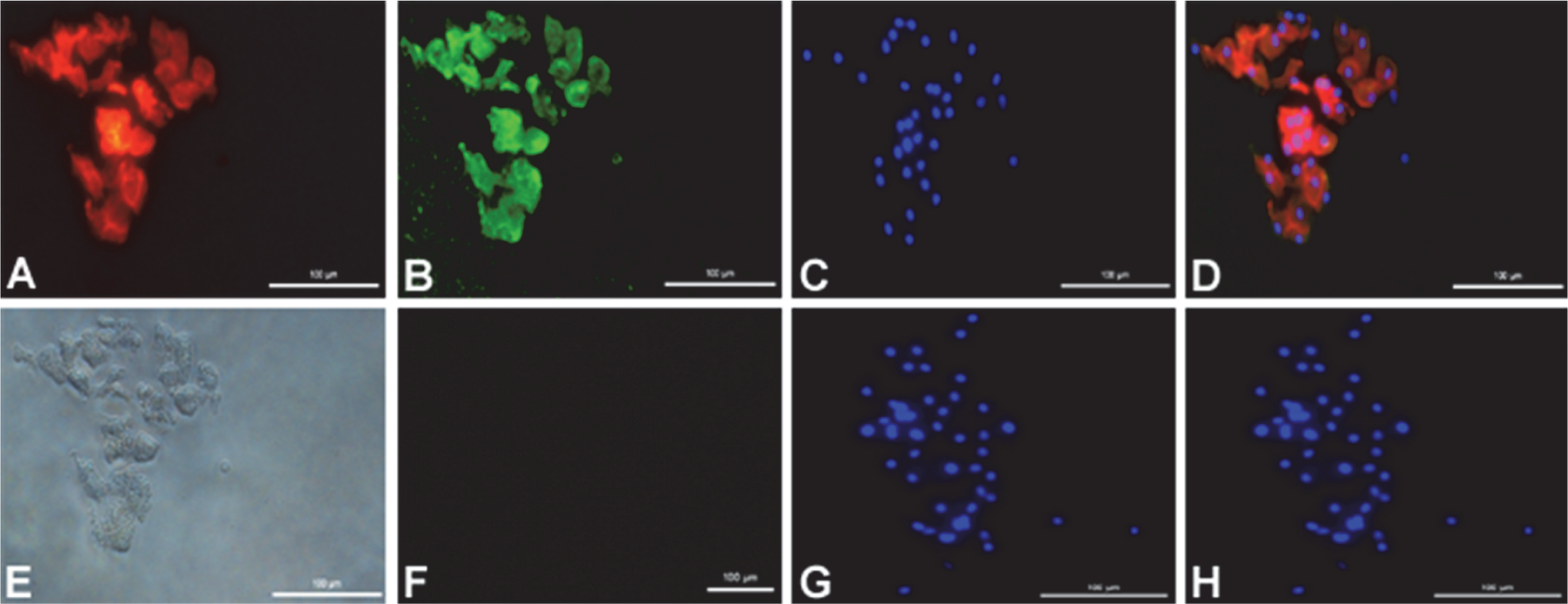
Immunostaining of 5 days old cultured buffalo hepatocytes with anti-cytokeratin-18 and anti-albumin. Immunostaining with (A) CY3 labelled anti-cytokeratin-18 antibodies (fluorescence signal in red); (B) FITC labelled anti-albumin antibodies (green); (C) staining of hepatocytes nuclei with DAPI (blue). Panel D shows the merged images from panels A, B and C. Panel E shows light microscopic image of hepatocytes; panel F shows negative control (Isotype control), and panels G shows staining with DAPI, while panel H shows images merged from panels F and G.

**Fig 6:**
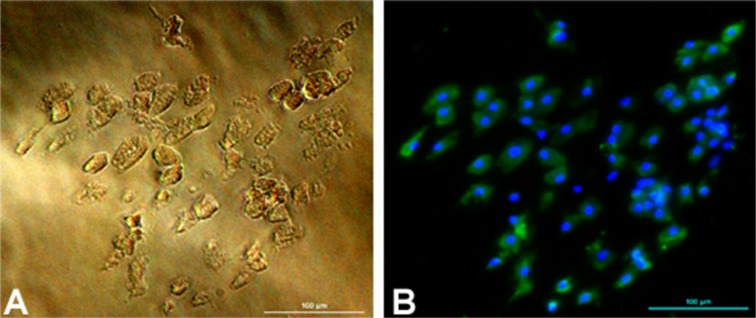
Immunostaining of 5 days old cultured buffalo hepatocytes with α1-antitrypsin antibody. Panel A shows light microscopic image of hepatocytes, and panel B shows immunostained hepatocytes with α1-antitrypsin antibody labelled with FITC (green) and nuclear stain DAPI (blue) dyes.

**Detection of hepatocyte-specific marker proteins by immunostaining**. Immunostaining with anti-cytokeratin-18 and anti-bovine serum albumin (anti-BSA) antibodies simultaneously revealed expression of cytoskeleton protein and albumin in the five days old cultured hepatocytes. Immunostaining with anti-cytokeratin-18, which is a cytoskeleton marker for intermediate filament, showed strong signal in all the cells. The intensely stained intermediate filaments were also observed. Strong signal with anti-BSA antibody in hepatocytes suggested high expression of albumin. No specific staining was observed in negative control experiment with mouse and rabbit IgG isotype (Fig. 5). Another hepatic protein α1-antitrypsin was also detected by immunostaining of the cultured cells (Fig. 6).

**Fig 7:**
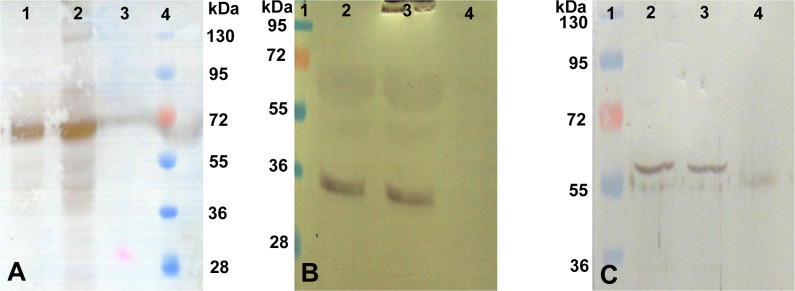
Western blot analysis of 5 days cultured buffalo hepatocytes. Blots of buffalo hepatocytes lysate by using antibodies against albumin (Panel A); lane 1: HepG2 (Positive control), Lane 2: buffalo hepatocytes (test); Lane 3: skin fibroblast (negative control); Lane 4: pre-stained protein marker; cytokeratin-18 (Panel B); Lane 1: pre-stained protein marker; Lane 2: HepG2 cells; Lane 3: buffalo hepatocytes; Lane 4: skin fibroblast; and α1-antitrypsin (Panel C), order of lanes is similar to that shown in panel B.

**Fig 8:**
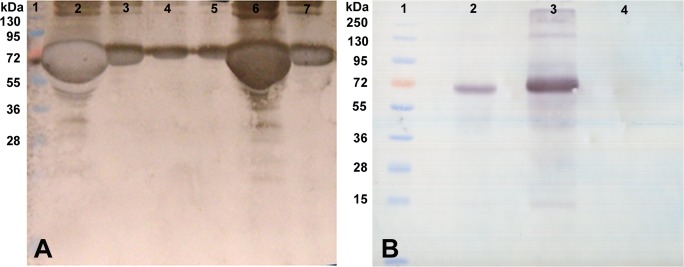
Temporal expression and secretion of albumin in condition media (CM) of hepatocytes. Panel A—Western blot analysis of albumin using condition media obtained from different time points of hepatocytes culture using FBS-free William’s E media. Lane 1: pre-stained protein marker; Lane 2: fresh hepatocytes lysate; Lane 3: 1^st^ day CM; Lane 4: 3^rd^ day CM; Lane 5: 4^th^ day CM; Lane 6: 5^th^ day CM; Lane 7: 7^th^ day CM. Panel B—Control experiment. Lane 1: pre-stained protein marker; Lane 2: pure hepatocytes (positive control); Lane 3: condition media (test); Lane 4: FBS-free William’s E media (negative control).

**Western blot analysis of hepatocyte-specific marker proteins**. Western blotting also suggested the expression of hepatocyte specific marker proteins including albumin, cytokeratin-18 and α1-antitrypsin in the cultured cells. Albumin was profusely expressed by the cultured hepatocytes. Skin fibroblasts used as negative control did not express albumin (Fig. 7). Western blot of cytokeratin-18, which constitutes the cytoskeleton of epithelium cells, showed good expression in the cultured as well as in the HepG2 cell line that was used as a positive control. The fibroblast cells used in the negative control did not show expression of cytokeratin-18 (Fig. 7). The temporal expression of albumin in condition media was detected by Western blot for up to seven days (Fig. 8). We observed that albumin secretion was highest in fresh hepatocytes. Thereafter it decreased gradually on 1^st^, 3^rd^, and 4^th^ day but again a sudden increase was observed on 5^th^ day followed by a decrease on 7^th^ day.

**Fig 9:**
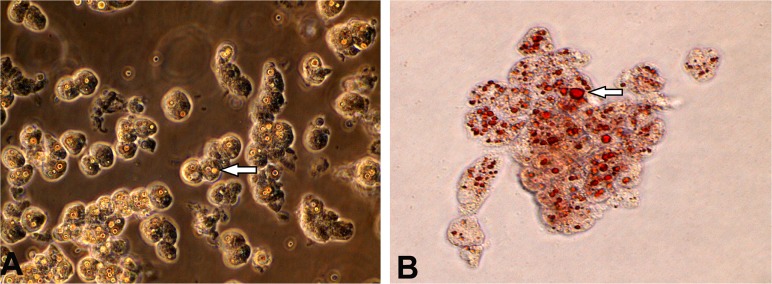
Oil-red staining of lipid droplets in 5 days old cultured buffalo hepatocytes. Panel A shows lipid droplets (indicated by arrow) in hepatocytes in phase contrast at 200X magnification; and panel B shows oil red stained hepatocytes containing lipid droplets (arrow).

**Detection of lipid droplets in hepatocytes**. In hepatocytes, triacylglycerol and cholesteryl esters are stored in lipid droplets and could be observed by staining cultured hepatocytes with oil red on fifth day (Fig. 9).

## Discussion

The objective of this study was to develop a convenient and cost effective procedure for isolation of functional buffalo hepatocytes and their short term *in vitro* culture. Increased use of veterinary drugs and growth promoting agents in livestock has led to accumulation of drug residues and their metabolites in the animal body and animal products like milk that could be hazardous for humans. The nitrogen and sulphur containing veterinary drugs generate toxic metabolites that could also affect animal health and productivity as well as human health. To study toxicological properties of drugs and their biotransformations in large ruminants, research attention has been focused on cattle hepatocytes [3, 16]. For isolating hepatocytes most of the studies used the Howard’s mechanical/enzymatic technique [11] modified by Berry and Friend [12] that was further improved by using two-step collagenase perfusion technique by Seglen [13]. Some other workers further modified Seglen’s method for hepatocytes isolation with high yield [18, 22]. But these methods required high collagenase concentration, extensive handling with perfusion and sieving apparatus.

We simplified the hepatocyte isolation technique by the combination of enzymatic perfusion with collagenase at several orders of magnitude lower concentration and mechanical disruption for high purity and yield of hepatocytes. We performed several key modifications in previously used isolation techniques. Instead of using expensive perfusion apparatus, we used 50 ml aseptic syringe for tissue perfusion first with EGTA to chelate Ca^2+^ and loosen the cell-cell interactions followed by second perfusion step with Ca^2+^ salt enriched collagenase type IV at a much reduced concentration to digest the tissue [17]. After this step, we performed additional mechanical disruption which accelerated disaggregation of the tissue. Bovine hepatocytes have been isolated only by mechanical disruption of liver tissue without any perfusion step; however that resulted in decreased cellular purity and required additional Percoll purification step [17]. Also, we used collagenase at much lower concentration at 37°C which required half the time for digestion of liver tissue as opposed to higher collagenase concentration at lower temperature. Extensive handling of hepatocytes for sieving was dispensed with and simple unit gravity sedimentation was used that saved on time and cost of the procedure. Washing the cells thrice by centrifugation at 50×g for a minute led to removal of RBC along with other cell types with the recovery of hepatocytes at 99% purity.

It is noteworthy that we could initiate second step of tissue perfusion with collagenase enzyme at least three hours after the first perfusion step because of transportation time required from the slaughter house to our laboratory. The initial period after removal of the tissue is most crucial and we believe that immediate completion of the procedure could significantly increase the yield and viability of hepatocytes.

Our results suggested reorganization of buffalo hepatocytes into clusters and restoration of cellular polarity and morphology after 24 hours of seeding on plates coated with ECM. However, BrdU incorporation assay suggested active growth on 3^rd^ day (seeding day was considered as day zero). The morphology of the isolated cells was well preserved. In fresh hepatocytes, the structural polarity of the cells was lost [12] and regained after cell growth in monolayer [23]. We observed typical polygonal configuration in sandwich culture system because it might provide effective three dimensional scaffold. In our study, hepatocytes multiplied for five days and survived longer on fibroblast feeder layer than on other ECMs. Reports suggest that hepatocytes cocultutred with rat liver epithelial cells [24, 25] could stay longer. Corlu et al. [26] described that rat liver epithelial cells provides cell-cell interaction and attachment factors for cell growth. Another report suggested use of mouse fibroblastic 3T3 cells in the coculture system [27] for culturing hepatocytes. In our case, using buffalo skin fibroblast cells as feeder layer for hepatocytes culture provided higher avidity of hepatocytes as well as enhanced life of hepatocytes, which could be because of growth and attachment factors like fibronectin, laminin and other extracellular components presented by fibroblasts. By using fibroblast feeder layers, hepatocytes could be grown for five days and maintained for up to nine to ten days in culture as opposed to other ECMs where cells faced apoptosis quickly and could not be maintained beyond seven days of culture.

We also carried out functional characterization of the cultured hepatocytes by studying the expression and detection of hepatocyte specific marker genes, proteins and enzymes as well as staining of fat droplets to show the functional viability of the isolated hepatocytes. The buffalo hepatocytes on 5^th^ day of culture expressed mRNA transcripts of genes encoding albumin, HNF-4α, glucose-6-phosphatase, tyrosine aminotransferase, and cytochromes P450 (CYP1A1 and CYP3A4). Albumin is an important marker for liver-specific function and most of the serum albumin is synthesized in liver [28]. We detected both transcript and protein level of albumin in the 5^th^ day cultured buffalo hepatocytes by RT-PCR and Western blotting, respectively. Immunocytochemical analysis revealed the presence of albumin in buffalo hepatocytes. Albumin was also detected in the condition media of cultured cells for up to seven days (Fig. 6), suggesting the secretion of albumin in the medium. The surge in albumin on 5^th^ day could be due to cell damage in culture condition, since the rise was again followed by decrease in albumin level. Several reports suggest albumin secretion in normal liver during first week after isolation [29–31]. In case of rat also, complete loss of albumin secretion in cultured hepatocytes was observed on 5^th^ day [18].

In cultured buffalo hepatocytes, real time PCR analysis revealed significant reduction in the albumin transcript level on 3^rd^ day of culture. Thereafter, the transcript level decreased gradually with culture time. We also observed gradual loss of secreted albumin except on 5^th^ day of culture (Fig. 6) that could be ascribed to loss of cellular architecture as well as cell membrane permeability. The decreased synthesis of albumin transcripts in cultured hepatocytes might result in the decreased albumin synthesis and secretion in condition media. Dunn et al. [32] also suggested transcriptional control on the rate of albumin production in cultured hepatocytes in single gel as well as sandwich system.

CYP1A1 and CYP3A4 genes, which encode cytochrome P450 and responsible for drug and xenobiotics metabolism, were also expressed on 5^th^ day of culture. However, in the 3–8 days old culture CYP1A1 expression decreased to half of that in fresh hepatocytes. CYP1A1 expression has been used to understand the functional viability of hepatocytes and reported to be downregulated in cultured rat hepatocytes [33]. These studies suggested the loss of functional activity because of loss of cellular polarity and cell architecture [34], since functional activity could be restored by culturing hepatocytes in the sandwich configuration [32, 34] or in the presence of increasing oxygen tension [35].

HNF-4α, an essential transcription factor for regulating the expression of several hepatic genes involved in lipid metabolism [36], was also detected in the isolated buffalo hepatocytes. Liver is the only organ where transamination reaction takes place, and it is also the storehouse of glycogen. Expression of tyrosine aminotransferase and glucose-6-phosphatase suggested that cultured buffalo hepatocytes are metabolically active for amino acid and glucose metabolism, respectively. Hepatocytes are epithelial cells with distinct apical and basal surface [18]. Abundant expression of epithelial cytokeratin-18 was observed particularly in the cellular periphery parallel to the cell surface in almost all the cells, which suggested the purity of hepatocytes population without contaminating cells like fibroblast, Ito and Kuffer cells. α1-antitrypsin synthesized in liver was also localized in the cultured hepatocytes. Triacylglycerol and cholesteryl esters are stored in lipid droplets and serve as important marker of hepatocytes. These lipids were also detected by oil red staining.

The expression of hepatocyte specific marker genes suggested the functional viability of the *in vitro* cultured hepatocytes as well as the suitability of the simplified isolation method for hepatocytes.

## Conclusion

Hepatocytes isolation by the modified protocol provided a fast and cost effective method for the isolation of pure and functionally viable hepatocytes with good yields for *in vitro* studies. The isolated cells expressed hepatocytes specific marker genes, proteins and enzymes and showed normal morphology when grown on ECM. Buffalo skin fibroblast feeder layer was observed to be the most effective support matrix that allowed buffalo hapatocyte culture for more than a week.

## Materials and Methods

### Reagents

Foetal bovine serum (FBS) was obtained from Hyclone (Canada). All other media and reagents unless otherwise specified were purchased from Indian supplier of Sigma Chemical Co. (St. Louis, MO).

### Ethics statement

Buffalo liver tissue was obtained from Ghazipur slaughter house, New Delhi, India with written request for use of sample for research purpose only. The liver sample was collected from the slaughtered animals and no animal was slaughtered specifically for the purpose of tissue collection.

### Hepatocytes isolation

The liver tissue was removed from the healthy animal immediately after the animal was slaughtered for meat purpose. For hepatocytes isolation only caudate lobe of liver was excised aseptically with surgical scalpel and rinsed for 30 seconds in 70% alcohol. Immediately after removal, liver sample was perfused with 500 ml of pre-cooled (4°C) Ca^2+^ and Mg^2+^ free 33 mM HEPES [4-(2-hydroxylethyl)-1-piperazineethanesulfonic acid] buffer (pH 7.6) containing 0.5 mM EGTA through the portal veins at a flow rate of 50 ml/ min by using a 50 ml sterile syringe fitted with 20 gauge needle. This helped to remove blood clot and loosen Ca^2+^ ion mediated cell-cell anchorage. Then another 250 ml of pre-cooled (4°C) 33 mM HEPES buffer at a flow rate of 25–30 ml/min was perfused and the liver sample was stored in 33 mM HEPES buffer in a sterile container. The processed buffalo liver sample was transported to laboratory in cool packs. It took around 3–4 hours to transport the sample to laboratory where the, liver tissue was placed in a sterile stainless steel-tray inside the bio-safety cabinet. The second step of perfusion was done at a flow rate of 50 ml/min with 100 ml of pre-warmed (37°C) Ca^2+^ ion enriched (using calcium chloride) 33 mM HEPES buffer (pH 7.6) containing collagenase type IV (500 ng/ml) and 0.125% hyaluronidase. The liver sample was then kept at 37°C for 10 minutes followed by mincing with sterile scalpel for 1–2 minutes in a 60×15 mm cell culture dish. Then pre-cooled (4°C) William’s E containing 5% FBS medium was added and the minced tissue were very slowly transferred to a sterile 50 ml falcon tube followed by incubation of sample on ice for 5 minutes to allow sedimentation of disaggregates at unit gravity. Supernatant was taken out gently and centrifuged at 100×g for a minute. The supernatant was discarded and cell pellet was washed thrice with William’s E containing 5% FBS medium by centrifugation at 50×g for a minute each time. Finally, cell pellet was resuspended in small volume of 10% William’s E medium supplemented with glutamine (5 mM), insulin (5 μg/ml), epidermal growth factor (10 μg/ml), hepatocyte growth factor (20 μg/ml), insulin like growth factor (10 μg/ml), 1X insulin transferrin selenium, albumin (BSA, Fraction V) (1 mg/ml), sodium pyruvate (10 mM), nicotinamide (10 mM) and 1% dimethyl sulfoxide.

### Assessment of cell yield and cell viability

Viability of hepatocytes was determined by trypan blue dye exclusion test [37]. 250 μl of diluted cell suspension and 100 μl of 0.4% trypan blue in phosphate-buffer saline was taken in a 1.5 ml microfuge tube and mixed gently. With a cover slip in place, small volume of trypan blue cell suspension was transferred to a counter chamber of the Haemocytometer. Viable (opaque) and non-viable (stained blue) cells were counted and percent cell viability and total cell yield per gram liver tissue were calculated.

### Short-term culture of hepatocytes

Unlike classical epithelial cells, hepatocytes have a belt of apical surfaces divided into two basolateral surfaces that are in contact with ECMs. Monolayer attachment culture *in vitro* condition using normal dishes is not suitable for cellular growth that restricts long-term culture with maintenance of physiological function of hepatocytes. Rather hepatocytes are well known for growing as monolayer or sandwich configuration on different types of ECM coated plates compared to normal polystyrene coated plates [38, 39]. The ECM promotes attachment and growth of hepatocytes with maintenance of liver-specific function. Matrigel containing collagens, laminin, fibronectin, tenascin, elastin, and a number of proteoglycans and glycosaminoglycans (MaxGel ECM, Sigma, USA), ECM gel from Engelbreth-Holm-Swarm mouse sarcoma (Sigma, USA), gelled collagen from bovine skin (Sigma, USA) and rat tail collagen I pre-coated tissue culture plates (Invitrogen, USA) were used as ECM for hepatocyte culture. Coating of different ECM was performed as per manufacturer protocol. Hepatocytes were seeded on different ECM configurations at a density of 1×10^5^ cells/cm^2^ in hepatocyte culture medium (William’s E) with 10% FBS and incubated at 37°C with 5% CO_2_ and 95% air.

### Culturing hepatocytes on fibroblast feeder layer

Adult skin fibroblast feeder layer was also used as a supportive matrix for hepatocytes culture to provide nutrition and attachment factor for hepatocytes growth. For preparation of adult fibroblast feeder layer, skin biopsies were taken from ear-pinna of adult buffalo and then washed 4–6 times with Dulbecco’s Phosphate Buffer Saline (DPBS) containing 10% FBS and 50 μg/ml gentamicin sulphate. The ear-pinna was cut into small pieces of 1 mm^3^ using sterile scissors and washed 3–4 times with DPBS containing 10% FBS and 50 μg/ml gentamicin sulphate and then with cell culture medium constituted of Dulbecco’s modified Eagle’s medium supplemented with 20% FBS and 50 μg/ml gentamicin sulphate. The tissue pieces were transferred into cell culture flask and cultured in the cell culture medium at 37°C with 5% CO_2_ and 95% air. After 6–8 days, when proliferation and establishment of primary fibroblast was observed, the explants were removed. After cell confluency reached, fibroblast monolayers were disaggregated and subcultured using 0.25% trypsin-EDTA. Feeder layer of fibroblast was prepared by using 10 μg/ml Mitomycin C treatment for four hours to arrest cell division (metaphase II) of growing cells. After four hours fibroblasts were washed twice with DPBS and sub-cultured in Dulbecco’s Modified Eagle’s Medium supplemented with 10% FBS and 50 μg/ml gentamicin sulphate onto the cell culture dishes at a seeding density of 5×10^4^ cells/cm^2^, resulting in the formation of feeder layer of adult fibroblast. Freshly isolated viable hepatocytes were seeded on the feeder layer at a cell density of 1×10^5^ cells/cm^2^. Medium was changed after four hours and then daily for seven days. Hepatocyte culture medium supplemented with 10% FBS was replenished with fresh medium at four hours after incubation of fresh hepatocytes at 37°C. Hepatocytes grown in different ECMs were routinely observed under inverted light microscope (Ti Eclipse, Nikon, Japan).

### BrdU incorporation assay

For determination of hepatocytes proliferation in culture, BrdU assay was performed as per manufacturer’s protocol using BrdU cell proliferation assay kit (Cat No. Q11A58, Calbiochem, USA). Hepatocytes were seeded on fibroblast feeder layer in a 96-well plate as 100 μl cells at the density of 100 cells/well. The growth of hepaocytes was assessed on 1^st^, 3^rd^, 5^th^, 7^th^ and 9^th^ day of culture by BrdU labelling. The BrdU incorporation was carried out by incubating BrdU reagent with growing hepatocytes for 2 hours followed by stopping reaction and measuring the absorbance at 540 nm on Nanoquant plate reader (Model: Infinite M200 PRO, TECAN). For the assay, hepatocytes from livers of three different buffaloes were isolated and each of them was seeded in triplicate resulting in nine measurements (*n* = 9) for each of the labelling experiment with feeder layer and without feeder layer on each day. All the three liver tissue samples were collected on the same day and processed in a similar manner and medium was replaced in all the wells till the day of BrdU assay. The data were represented as mean ± SE. A cell proliferation curve was prepared by plotting the absorbance of BrdU-lebelled hepatocytes against respective days of culture.

### Analysis of gene expression

Gene expression analysis of isolated hepatocytes was performed using RT-PCR. Total RNA was extracted by TRIzol method [40]. Confluent monolayer at 5^th^ day of hepatocytes culture was prepared by discarding the culture medium and rinsing twice with PBS. For RNA isolation, hepatocytes were harvested by using TRIzol reagent (Invitrogen, USA). 1 ml TRIzol reagent was used for each well of six well plate. Then chloroform was added and mixture was vortexed for 15 seconds followed by incubation at room temperature for 5 minutes. The sample was centrifuged at 12,000×g for 15 minutes at 4°C. The aqueous phase was transferred to a fresh microfuge tube and 0.5 ml of isopropanaol was added and kept at -20°C for 45 minutes. The sample was centrifuged at 12,000×g for 30 minutes at 4°C. The supernatant was removed and RNA pellet was washed with 1 ml 75% ethanol and centrifuged at 7,500×g for 5 minutes at 4°C. The RNA pellet was dried and dissolved in nuclease free water and stored at -80°C.

Prior to reverse transcription contaminating DNA if any in the RNA preparation was removed by treating with DNA-free kit as per manufacturer protocol (Ambion, Life Technologies). For DNase treatment, 1 μl rDNase I (2 U) was used for up to 10 μg RNA in a 50 μl reaction. Reverse transcription of mRNA was performed from 1 μg of total RNA in the presence of RNase inhibitor RNAguard (40 U/μl), oligo-dT-15 primers, deoxynucleotides (dNTP 10 mM), Mo-MuLV reverse transcriptase (200 U/μl) and the reverse transcriptase buffer in a 150 μl final reaction volume. Reverse transcription was performed at 42°C for 1 hour. All the components used in RT-PCR were procured from Fermentas, Lithuania.

Following gene-specific primers were designed by using Primer3 program for the amplification of cDNA fragments corresponding to coding segments of genes: albumin forward 5’-GGGGTGTGTTTCGTCGAGAT-3’ and reverse: 5’-CTCACAGCAGTCAGCCATGT-3’; tyrosine aminotransferase forward 5’-CGATTGGGGACCCTACTGT-3’ and reverse 5’- AAGCAACTTCCTCCCGACTG-3’; glucose-6-phosphatase forward 5’ GAGTCTTGTCAGGCATTGCG 3’ and reverse 5’ TCTTGAGGAGGCTGGCAAAG 3’; hepatocyte nuclear factor 4 alpha forward 5’-CTGGCAGAGATGAGTCGA-3’ and reverse 5’-CCTTGGCATCTGGGTCAA-3’; CYP1A1 forward 5’-CGACACTCCTCCTTTGTCC-3’ and reverse 5’- AGAGCTTCTGGTCATGG-3’; CYP3A4 forward 5’- CGATCCCTTTCTTCTCGCAGT-3’ and reverse 5’- GTCCACACGTGGCTTTTGA-3’. The glyceraldehyde 3 phosphate dehydrogenase (GAPDH) gene was used as positive control in all the RT-PCR assays by using the primer pair forward: 5’-CCAAGGTCATCCATGACAACTTTG-3’ and reverse: 5’-GGTCCACCACCCTGTTGCTGTAG-3’.

Each primer pair was designed in such a way that primers were located either in different exons or spanned exon-exon junction: albumin forward in exon 1and reverse in exon 4; tyrosine aminotransferase forward spanning exon 2-exon3 junction and reverse in exon4; glucose 6 phosphatase forward spanning exon 4-exon5 junction and reverse in exon5; HNF-4α forward in exon 8 and reverse in exon 9; CYP1A1 forward in exon5 and reverse spanning exon 6-exon 7 junction; CYP3A4 forward in exon7 and reverse spanning exon 8-exon 9 junction. PCR comprised of 35 cycles, each cycle consisting of a denaturing step at 95°C for 1 minute, a primer annealing step (at 58–60°C, depending on the primer pair) for 1 minute and a primer extension step at 72°C for 1 minute. The reactions were preceded by a first denaturing step of 3 minutes at 95°C and followed by a final elongation step of 5 minutes at 72°C.

### Quantitative RT-PCR of hepatocyte-specific genes

Relative quantification of albumin and CYP1A1 genes was performed by using Lightcycler 480 SYBR green I Master Mix (2X) (Roche) in Light Cycler 4800 Real-Time PCR system (Roche). The housekeeping genes β-actin and GAPDH were used as reference genes for normalization of target genes expressed in buffalo hepatocytes. The same sets of primer pairs for albumin, CYP1A1and GAPDH described in previous section were used for qPCR analysis of gene expression. For β-actin gene, the primer sequences with forward 5'-AGAAAATCTGGCACCACACC-3' and reverse 5'-GTCAGGCAGCTCGTAGCTCT-3' sequences were used. The specificity of the pair of primers was checked by melting curves of the amplified products. Temporal expression of albumin and CYP1A1 genes was determined in fresh and cultured buffalo hepatocytes from three different animals on 3^rd^, 5^th^ and 8^th^ day of culture.

### Immunostaining of hepatocytes

For immunostaining of hepatocytes and Western blotting, antibodies against BSA, cytokeratin-18 and α1-antitrypsin were procured from Sigma Chemical Company. Hepatocytes were fixed with 5% paraformaldehyde into Phosphate Buffer Saline (PBS) for 30 minutes at room temperature. Cells were then permeabilized by incubation with 0.2% Triton X-100 in PBS for 10 minutes. Hepatocytes were washed thrice with PBS and then incubated for one hour with NAP blocker (G Biosciences, USA) in PBS with 1:2 ratio for blocking the hepatocytes. Hepatocytes were incubated with the primary antibody in the blocking solution for 60 minutes at room temperature. Hepatocytes were washed thrice for 5 minute each time in PBS and then incubated with the secondary antibody for 30 minutes at room temperature in darkness. FITC-conjugated anti-rabbit IgG for the detection of BSA; Cy3 labelled anti-mouse IgG for the detection of cytokeratin-18 and FITC-conjugated anti-mouse IgG for the detection of α1-antitrypsin were used as secondary antibodies. Hepatocytes were washed thrice with Tris-Buffered Saline and Tween 80: 20 mM Tris base containing 137 mM NaCl and 0.1% Tween 20 (TBST) and incubated for 2 minutes with DNA staining with 300 nM DAPI dye for counterstaining of nucleus. Finally, after another three washes in PBS, hepatocytes were observed under the phase contrast and fluorescence microscope (Ti Eclipse, Nikon, Japan) with appropriate excitation and emission filters.

### Western blot analysis of marker proteins

Confluent monolayer culture of hepatocytes was prepared by discarding the culture medium and rinsing twice with PBS. Then hepatocytes were harvested by cell scrapper and resuspended into PBS in a 15 ml falcon tubes. After brief centrifugation at 500×g for 1 minute, hepatocytes were left for 2 minutes for sedimentation under gravity. Supernatant was discarded and total protein was isolated from buffalo hepatocytes using Qproteome mammalian protein isolation kit (Qiagen, USA) as per manufacturer protocol. Total protein concentration in hepatocytes lysate was quantified by Bradford assay [41]. 50 μg of proteins from each sample were separated by electrophoresis on 12% SDS-PAGE followed by 5 minutes incubation of gel in Transfer Buffer (24 mM Tris base containing 194 mM glycine and 10% methanol). The gel was transferred to methanol activated 0.45 μm PVDF Transfer Membrane (MDI, India) by using semi-dry transblot apparatus (GE Biosceinces, USA) for 1 hour. In next step, after 1 hour incubation of membrane in the NAP blocker (G Biosciences, USA) in TBST at 37°C, membranes were incubated with primary antibodies against hepatocyte specific marker proteins like bovine serum albumin (1:5000), cytokeratin-18 and anti-α1-antitrypsin (1:2000) and GAPDH (control) diluted in TBST and incubated overnight at 4°C. Three washes with TBST were followed by the corresponding secondary antibody incubation for 2 hours at 37°C. The secondary antibody, HRP-conjugated anti-mouse or anti-rabbit IgG, was used at 1:1000 dilution. HRP-conjugated anti-rabbit IgG for BSA, and HRP-conjugated anti-mouse IgG for cytokeratin-18 and α1-antitrypsin were used as secondary antibodies for detection of respective proteins. The peroxidase substrate 3,3-diaminobenzidine (DAB) (Merck India) was used to develop the blot. Cultured HepG2 cells and bovine adult fibroblasts used as positive and negative control for western-blot analysis, respectively, were also given similar treatment. To assess the temporal expression of albumin in buffalo hepatocytes, we performed western-blot analysis of condition media collected on 1^st^, 3^rd^, 4^th^, 5^th^ and 7^th^ day of hepatocyte culture. For this experiment, hepatocytes were separately grown on dry collagen-coated dishes using FBS-free William’s E media for 7 days. However for initial attachment, 5% FBS was used for first 4 hours after seeding of fresh hepatocytes. On the respective day hepatocytes culture medium was aspirated and centrifuged at 1000×g for 5 minutes. The supernatant (condition media) was collected and used for analysis.

### Oil red staining of lipids in hepatocytes

Oil red staining was performed to visualize the lipid droplets in hepatocytes. Medium was removed from monolayer hepatocytes culture. 10% formalin was added and incubated for 5 minutes at room temperature. The formalin was discarded and fresh formalin was added and further incubated for 1 hour. Formalin was removed and washed with 60% isopropanol and allowed to dry completely under laminar flow. Working oil red stain (mixing of stock oil red and water in 3:2 ratio) was added and incubated for 10 minutes. Then oil red stain was removed and washed with distilled water for four times and observed under inverted microscope.

### Statistical analysis

The data on liver processing, hepatocyte isolation, cell viability and purity were obtained from at least three independent hepatocyte isolations at different times from tissue collected from different animals or as mentioned in the corresponding sections. The data on cell yield, viability and cell purity has been presented as mean ± standard error of mean (SEM), analysed from three different experiments. In each experiment, number of cells in at least three microscopic fields of view were counted and analysed. The expression data on each of the marker gene or protein was collected by 3–6 trials from at least three independent cultures. Students’ paired t-test was performed in Microsoft Office Excel 2007 for comparison of albumin and CYP1A1 gene expression in hepatocytes at different days of culture.
